# Illegal interlocks among life science company boards of directors

**DOI:** 10.1093/jlb/lsae005

**Published:** 2024-04-13

**Authors:** Anoop Manjunath, Nathan Kahrobai, Mark A Lemley, Ishan Kumar

**Affiliations:** Stanford University School of Medicine, Stanford University, Stanford, CA, USA; Stanford Law School, Stanford, CA, USA; Goodwin Procter LLP, Santa Monica, CA, USA; Stanford Law School, Stanford, CA, USA; Stanford University School of Medicine, Stanford University, Stanford, CA, USA; Stanford Law School, Stanford, CA, USA; Institute for Stem Cell Biology and Regenerative Medicine, Stanford University School of Medicine, Stanford, CA, USA

**Keywords:** antitrust, biotechnology, life sciences, interlocking directorates, competition

## Abstract

Competition between life science companies is critical to ensure innovative therapies are efficiently developed. Anticompetitive behavior may harm scientific progress and, ultimately, patients. One well-established category of anticompetitive behavior is the ‘interlocking directorate’. It is illegal for companies’ directors to ‘interlock’ by also serving on the boards of competitors. We evaluated overlaps in the board membership of 2,241 public life science companies since 2000. We show that a robust network of interlocking companies is present among these firms. At any given time, 10–20 percent of board members are interlocked; the number of interlocks has more than doubled in the last two decades. Over half of these interlocked firms report over $5 million in historical revenue, exceeding a legal threshold that makes an interlocking directorate a violation of antitrust law. Those interlocks are only illegal if the companies compete, even in part. Using the disease categories for which companies have sponsored clinical trials, we discover that a few markets are responsible for a large fraction of interlocks. We show that in dozens of cases, companies share directors with the very firms they identify as their biggest competitive threats. We provide a data-driven roadmap for policymakers, regulators, and companies to further investigate the contribution of anticompetitive behavior to increased healthcare costs and to enforce the law against illegal interlocks between firms.

## I. INTRODUCTION

Competition between companies is critical to ensure that consumers receive the best possible product at the lowest possible price.^1^^,^^2^ However, the U.S. life sciences industry has recently come under public and regulatory scrutiny for a prevailing market dynamic in which healthcare costs, drug prices, and corporate profits are all rising.^3–5^ Is the life sciences industry in fact competitive? This question’s importance to patients and the healthcare system was brought into focus in a recent landmark antitrust case in which the U.S. Federal Trade Commission said that after a merger between Illumina, a leader in sequencing technology, and Grail, a multi-cancer detection company which relies on sequencing cell-free DNA, ‘Illumina will control the fate of every potential rival to Grail for the foreseeable future… [causing] substantial harm to U.S. consumers, who would experience reduced innovation, as well as potentially higher costs and reduced choice and quality for these life-saving products’.^6^

Antitrust law is designed to reduce prices and encourage innovation by ensuring that companies compete rather than collude. Since 1914, the Clayton Antitrust Act has made it unlawful for competitors to share directors (and since 1990, to share officers).^7^^,^^8^ This rule contains exemptions only for companies that have less than $4.1 million in competitive sales or where the competitive overlap between the companies is less than 2% of their sales. Interlocking officers and directors between companies that compete, even in part, are illegal *per se—*without any inquiry into whether the companies in fact restrained competition because of their overlapping interests or whether the conduct offered procompetitive benefits.^9–11^ One rationale for this rule is to prevent conflicts of interest, since officers and directors have fiduciary responsibilities to their corporations, and having responsibilities to competing companies is likely to prevent them from competing vigorously.^12^ Another is to reduce the risk that competitors coordinate their pricing and product decisions.

Within a market, interlocked directorates may form for a variety of reasons, including relationships between individuals (past personal interactions in society or business) and necessity (a limited supply of qualified individuals).^13^ To some extent, these factors are linked; individuals qualified to be board members often gain the requisite expertise at companies in the same line of business.^14^^,^^15^ Interlocking directorates may be a feature of the life sciences industry because its companies require officers and board members with the rare domain expertise required to usher cutting-edge, disease-specific products through clinical trials and into the market. Interlocked directorates are a measure of how concentrated socio-economic influence is among companies. Their varying prevalence across geographic regions may be reflective of cultural differences among business communities.^16–22^ Nonetheless, interlocks pose real risks to competition. An interlocked board member may encounter conflicts of interest because directors engage in documented meetings at regular intervals and have influence over corporate behavior at each company they help oversee—giving them the needed information and opportunity to make decisions that ultimately restrain competition between their companies. A high-profile example involves Google, whose CEO sat on the board of Apple until the Federal Trade Commission (FTC) intervened in 2009, despite the fact that the two companies are the largest makers of smartphone operating systems.^23^ Interlocks also provide opportunities for firms to pursue and conceal cartels. Companies with interlocked boards have been shown to act in parallel more and share knowledge among themselves.^24–28^

Although they were a subject of significant attention in the 1950s, interlocking directorates have received little government or scholarly attention in recent decades.^29^ But that may be changing. In the past few years, both the Antitrust Division of the U.S. Department of Justice and the Federal Trade Commission called attention to the issue and brought actions against companies with illegal interlocks, forcing the compromised directors to resign.^30–32^

## II. STUDY DESIGN AND RESULTS

**Figure 1:**
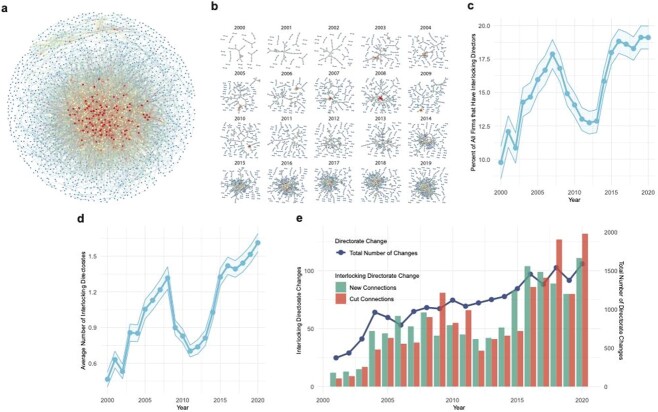
Interlocking directorates across all public life sciences companies from 2000 to 2020. (**A**) Connectome of all public companies included in the dataset from the years 2000–20. Companies (nodes) were linked if they shared a board member at any point in time (edges). Cooler colors indicate companies with fewer interlocking directors, warmer colors represent companies with greater numbers of interlocking directors. (n = 2241) (**B**) Time-series of the connectome in (A), annually from 2000 to 2019. (**C**) Percent of all companies that have interlocking directors annually from 2000 to 2020 (**D**) Average number of interlocking directorates for all companies in the dataset annually from 2000 to 2020. (**E**) Number of interlocking directorates formed and dissolved between companies (left y-axis) and total number of directorship changes (right y-axis) annually from 2001 to 2020.

To investigate the extent of interlocks in the industry, we developed a novel dataset linking the corporate boards and clinical trial portfolios of 2241 life sciences companies that have been publicly traded at any point in the United States since 2000. We connected the officers and boards of directors (‘directorates’) of all U.S.-listed firms that have filed a 10 K financial report and have a Standard Industrial Classification code administered by the SEC’s Office of Life Sciences, broadly including the biotechnology and pharmaceutical industries. We built an index linking these firms to U.S. clinical trials they have sponsored. The resulting 2241 firms were graphically connected based on whether they had shared board members (ie ‘directors’) at any point in time between 2000 and 2020, generating a ‘connectome’ to determine whether interlocking directorates are a historical feature of the life sciences industry (Fig. 1A). This revealed extensive connectivity between firms, with 6.19 average connections and 4 median connections per node (Fig. S1A). Time analysis from 2000 to 2020 revealed that the network has steadily increased in density and complexity over time (with the exception of the Great Recession period) (Fig. 1B). At any given time, between 10 and 20% of biotech companies have interlocked directorates (Fig. 1C). During the 20 years of our study, the average number of interlocking directorates per company grew threefold, from 0.5 to 1.7 (Fig. 1D). Interlocking directorates formed and dissolved almost 10 times as frequently in 2020 as they did in 2000, compared to a near-fourfold increase in board member turnover generally in the same period (Fig. 1E, Fig. S1C). Furthermore, interlocked directors’ average tenure was 37.9% longer than that of non-interlocking board members of an interlocked board and 50.5% longer than that of board members on non-interlocked boards (Fig. S1B). Interlocked directors thus remain on boards longer than their non-interlocked peers; these data reveal that the legal definition of interlocking directorates in fact reflects an observable subpopulation of life sciences directors with distinctive professional dynamics.

**Figure 2:**
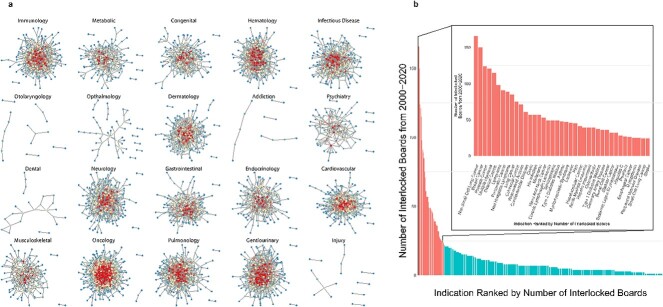
Interlocking directorates of companies that have sponsored clinical trials for indications within a given disease category. (**A**) Connectomes of companies with interlocking directorates between 2000 and 2020 by the disease categories of clinical trial indications they sponsored. Cooler colors indicate companies with fewer interlocking directors, warmer colors represent companies with greater numbers of interlocking directors. (**B**) Disease indications ranked by the number of interlocked companies that have sponsored clinical trials in these diseases, from 2000 to 2020. The inset shows the top 25 disease indications.

We analyzed how interlocking directorates influence companies’ participation in specific product categories (ie markets) by subdividing interlocked companies according to the disease spaces of the indications targeted by their interventional clinical trials. We discovered that interlocking directorates are prevalent among companies developing products related to oncology, immunology, hematology, neurology, and genitourinary diseases, and are present to a lesser extent in psychiatry, dental, and ophthalmology (Fig. 2A). The dynamics of interlocked boards are similar between these categories (Figs S2 and S3).

Interlocked directorates’ incentives to restrain competition are heightened in those cases where companies are operating in the same disease space. However, identifying whether two companies are actual competitors for the purposes of the antitrust law requires case-by-case analysis, and in many cases is eventually determined through litigation.^33^ Although that case-by-case analysis is not possible in a large-scale study of this sort, we tested the robustness of our overall findings by identifying two narrower and more targeted definitions of competition.

First, we considered companies that are pursuing interventional clinical trials for the same disease indication to be competitors, because interventions being tested in clinical trials are benchmarked against the standard of care (this definition is inclusive of the sub-characteristics of a therapeutic, such as modality, delivery, etc., which may also constitute improvements over the standard of care). This is a narrower, more precise definition than industry subgroup. For example, two companies making cardiology drugs might not compete directly if the drugs target different diseases. But companies developing and marketing drugs targeting the same disease indication are much more likely to be competitors. Using this narrower definition, we nonetheless find hundreds of instances of companies producing directly competitive drugs that share board members, and we find many disease indications that have dozens of interlocks (Fig. 2B, Table S1). This result is particularly strong in certain disease spaces, and is observed across a wide range of disease indications. Thus, our data show that even at the narrower disease indication level, many companies have interlocked boards with direct competitors. Furthermore, interlocked board members’ dynamics do not vary significantly by disease space, suggesting that these dynamics are ubiquitous and offering reason to believe they may be largely homogenous across the broader life science industry.

Second, the narrowest and most stringent method of identifying competitors is when companies self-report their competitors in their regulatory filings. This is likely to significantly undercount illegal interlocks, because companies are not required to self-report at all, because if they do report they are likely to limit reporting only to one or two companies they view as the most significant competitive threat, and because of their strong incentives to under-report potentially illegal interlocks with a competitor. Thus, we view self-reported interlocks with competitors in a public filing to be a lower bound on the number of illegal interlocks. We found a low, basal level of interlocking every year even among self-reported competitors (Fig. 4). However, we emphasize that this method does not capture the far more numerous interlocks between potential (unreported) competitors.

**Figure 3:**
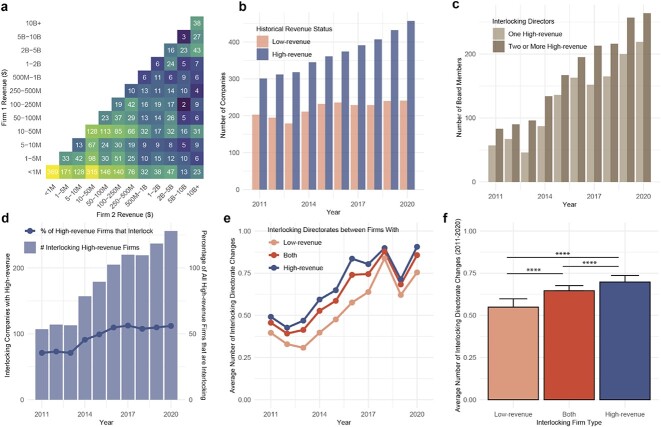
Interlocking directorates between companies based on lifetime revenue. (**A**) Heatmap of companies interlocked by historical revenue brackets from 2011 to 2020, on a year-by-year basis. The value in each tile represents the number of instances two companies with given revenue ranges have interlocked. Warmer colors indicate more instances of interlocking; cooler colors indicate fewer instances. (**B**) Number of companies annually reporting greater than or less than (or equal to) $5 million in lifetime revenue between 2011 and 2020. (**C**) Number of interlocked board members of companies annually reporting greater than or less than (or equal to) $5 million in lifetime revenue between 2011 and 2020. (**D**) Number of companies with greater than $5 million in historical revenue that also have interlocking directorates (left y-axis). Companies with greater than $5 million in historical revenue that also have interlocking directorates, as a percentage of all companies with greater than $5 million in historical revenue (right y-axis). (**E**) Average number of board changes for companies with greater than or less than $5 million in historical revenue from 2011 to 2020. (**F**) Average number of changes in interlocking directorates based on historical revenue, from 2011 to 2020.

Biotechnology companies sometimes spend years and even go public before generating revenue. Interlocking directorates are illegal if they are between companies that have competitive revenue exceeding a minimum threshold ($3.74 million in 2022).^34^^,^^35^ We characterized all pairs of interlocked companies based on their historical revenue (Fig. 3A). To be conservative and to add a stringent cutoff for the purposes of this analysis, we identified companies that had more than $5 million in historical revenue (‘high-revenue companies’) and less than $5 million in revenue (‘low-revenue companies’). But we note that many biotechnology companies are pre-revenue, and they are not included in the scope of current law even if their directors interlock. Similarly, companies with revenue from unrelated areas but no overlapping revenue do not fall under current law.

**Figure 4:**
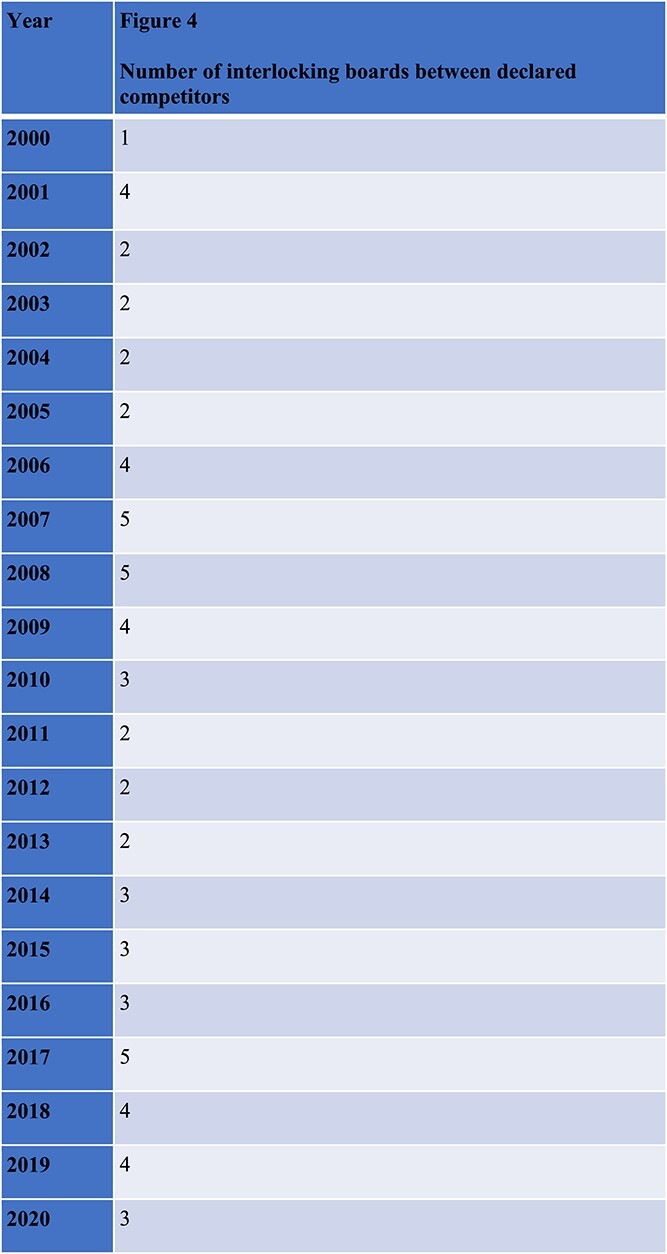
The number of companies each year that shared an officer or director with a company that they described in securities filings as one of their most significant competitors.

Companies with less than $1 million in revenue are highly interlocked with each other and with companies earning greater revenue more generally, but those companies are likely not covered by current law. Higher revenue companies (eg with $10 m–$50 m in revenue) also exhibit high levels of interlocking with lower and higher revenue companies (from <$1 m to $500 m in revenue). Companies with the highest revenues (>$2b) have increased interlocks with each other. Interlocks are observed between all possible revenue pairs.

An increasing proportion of companies became high revenue companies since 2011 (the earliest date for which consistent data are available) (Fig. 3B). The volume of interlocked board members of high-revenue companies—those to whom the law most likely applies—has grown steadily since 2011, and interlocked board members tend to be on more than one high-revenue company’s board (Fig. 3C). Over the course of the past decade, the number of interlocking companies that satisfy the Clayton Antitrust Act’s revenue threshold has more than doubled, and now represents over half of all high-revenue companies—an increase from 107 companies in 2011 to 256 companies in 2020 (Fig. 3D).

Although overall board membership turnover is largely similar between high- and low-revenue companies, interlocked board members who are on the boards of low-revenue companies have changed less frequently on an annual basis (Fig. 3E). In aggregate, low-revenue companies’ interlocked directors are changed an average of 0.55 times per year, while high-revenue companies’ interlocked directors are changed an average of 0.70 times per year, a 27% difference (Fig. 3F). Interlocked directors at low-revenue companies have slower turnover; this added staying power gives them more opportunity to influence company behavior. Anticompetitive behavior in these smaller companies is a particular concern because they sponsor early-stage drug development and sustain a diverse portfolio of experimental therapeutics.

## III. DISCUSSION

Across the life sciences sector, a significant proportion of all life sciences companies, and over half of all high-revenue companies, are currently interlocked with actual or likely competitors. Many of those companies share directors even as they pursue new approval for drugs in direct competition with each other. The high-revenue companies’ interlocked boards within particular disease spaces and specific clinical trial indications are the ones most likely to meet the Clayton Act’s definition of anticompetitive behavior.

Taken together, we find that a significant fraction of life sciences companies (and in some disease spaces, a majority) appear to be potentially out of compliance with federal antitrust law. Life sciences companies regularly employ officers or directors who have a conflicting relationship with actual or likely competitors. These interlocked board members stay on boards longer than their non-interlocked peers, potentially giving them greater opportunities to influence and align competitors’ behavior. Concerningly, the occurrence of interlocked boards within several major disease spaces suggests that interlocking boards may be part of normal business practice in the life science industry. Indeed, half of all high-revenue companies are interlocked; anticompetitive behavior among them is likely to result in greater direct economic impact on consumers. However, extensive interlocking between low-revenue companies may reduce the variety of drugs being tested for efficacy, which would have longer term effects on consumers. These effects may manifest in fewer therapeutic avenues available to patients, increased drug prices, and fewer efficacious drugs developed. These results highlight the importance of further investigation into the impact of interlocked boards on consumers and data-driven studies into how antitrust concerns influence drug development.

Our study has several limitations that may be explored in future research. Our dataset is limited to publicly traded companies within the United States. It is possible that interlocked directorates are still more prevalent among startups and other private companies, since many of those companies are funded through a small network of venture capital firms that tend to place their own employees as officers and directors of the companies they fund.^36^ Global firms outside of the U.S. (and thus not considered in our analysis) may have different dynamics that are specific to their home markets. In addition to product development, other forms of corporate behavior may be affected by interlocked directorates, such as litigation, licensing, and IP generation.

Our findings open several avenues for future research. Comparison of the connectomes of the life sciences industry to connectomes in other technology-related sectors may help explain whether the high degree of connectivity between firms in life sciences is unique to the sector or part of broader business trends and practices. Bottom-up analysis of companies’ drug portfolios would shed light on whether interlocked boards influence the novelty, variety, and risk-tolerance of drug development programs. And an investigation into the background of each director may reveal whether the directors are promoted by investors or come from a scientific or academic background. It may also expose additional indirect interlocks in which the same investor places its own employees on competing boards. We hope to explore those firm-level interlocks in future research. Many biotechnology companies are pre-IPO. Our data reaches only publicly traded companies, but we hope in future research to extend our work to private companies.

Our findings also have potential implications for public policy. Interlocked companies may be more likely to engage in cartels or other anticompetitive behavior such as pay for delay settlements, just as prior research has shown that companies with interests in both branded and generic drugs compete less vigorously as generics.^37^ The highly regulated nature of the biotechnology industry means that companies frequently plan to compete in an industry years before they actually enter the market and generate revenue. But pre-revenue interlocks may have many of the same competitive harms as the ones the law currently prohibits. So one implication of our work may be that the law should extend to companies that are in ‘nascent competition’—they do not yet have revenue in a market but have indicated an intent to enter the market. Companies which are in nascent competition may perhaps be defined as companies which are in Phase 3 trials in the same indication, simultaneously.

Finally, a more detailed investigation of competition between these firms may shed light on the wisdom and efficacy of antitrust law. It is surprising that there is such a large disconnect between what the law requires and what companies do. That may indicate ignorance of (or disregard for) the law, but it may also cause us to rethink a law that diverges so far from industry practice.

## Supplementary Material

Supplementary_data_lsae005

## Data Availability

The SEC filings containing board of director information utilized in this study are available at https://www.sec.gov/Archives/edgar/daily-index/bulkdata/submissions.zip. The code and data are available at https://github.com/amanjuna/directorates.

## Footnotes

1 L.M. Khan, *Amazon’s Antitrust Paradox*, Yale Law J. 126, 710 (2017).

2 H. Hovenkamp, *The Slogans and Goals of Antitrust Law*, SSRN, http://dx.doi.org/10.2139/ssrn.4121866 (2022).

3 A. Weber, L. Lin & M. Mrkaic, U.S. Healthcare: A Story of Rising Market Power, Barriers to Entry, and Supply Constraints. https://ssrn.com/abstract=4026379. IMF Working Paper No. 2021/180 (2021).

4 N.U. Sukhatme & M.G. Bloche, *Health Care Costs and the Arc of Innovation*, Minnesota Law Rev. (2019).

5 Policy Statement of the Federal Trade Commission on Rebates and Fees in Exchange for Excluding Lower-Cost Drug Products, Federal Trade Commission (2022).

6 Complaint ¶ 13–14, Illumina, Inc. and Grail, Inc., FTC Docket No. 9401 (March 30, 2021).

7 Clayton Antitrust Act §8, 15 U.S.C. §19.

8 FTC Announces Annual Update of Size of Transaction Thresholds for Premerger Notification Filings and Interlocking Directorates, Federal Trade Commission, https://www.ftc.gov/news-events/news/press-releases/2022/01/ftc-announces-annual-update-size-transaction-thresholds-premerger-notification-filings-interlocking (2022).

9 Protectoseal Co. v. Barancik, 484 F.2d. 585, 589 (7^th^ Cir. 1973).

10 Jicarilla Apache Tribe v. Supron Energy Corp., 782 F.2d 855 (10^th^ Cir. 1986).

11 United States v. Sears, Roebuck & Co., 111 F. Supp. 614 (S.D.N.Y. 1953).

12
P.E. Areeda & H. Hovenkamp, Antitrust Law: An Analysis of Antitrust Principles and Their Application ¶1300 (4th ed. 2016).

13 M.S. Mizruchi, *What Do Interlocks Do? An Analysis, Critique, and Assessment of Research on Interlocking Directorates*, Ann. Rev. Soc. (1996).

14
*Id.*

15 P.C. Dooley, *The Interlocking Directorate*, Am. Econ. Rev. (1969).

16 N. de Graaff & D. Valeeva, *Emerging Sino-European Corporate Elite Networks*, Dev Change (2021).

17 J. Koskinen & C. Edling, *Modelling the evolution of a bi-partite network Peer referral in interlocking directorates*, Soc. Netw. (2012).

18 N. Friel et al., *Interlocking directorates in Irish companies using a latent space model for bipartite networks*, Proc. Natl. Acad. Sci. U S A (2016).

19 W. Carroll et al., *The Corporate Elite and the Architecture of Climate Change Denial: A Network Analysis of Carbon Capital’s Reach into Civil Society*, Can. Rev. Sociol. (2018).

20 E.M. Heemskerk, F. Daolio & M. Tomassini, *The Community Structure of the European Network of Interlocking Directorates 2005–2010*, PLoS One (2013).

21 K. Van Veen & J. Kratzer, *National and International Interlocking Directorates Within Europe: Corporate Networks Within and Among Fifteen European Countries*, Econ. Soc. (2011).

22 A.G. Larsen & H.E. Christoph, *Identifying Power Elites—k-Cores in Heterogeneous Affiliation Networks*, Soc. Netw. (2017).

23
https://www.ftc.gov/news-events/news/press-releases/2009/08/statement-bureau-competition-director-richard-feinstein-regarding-announcement-google-ceo-eric (accessed Mar. 24, 2024).

24 D. Westra et al., *Understanding Competition Between Healthcare Providers: Introducing An Intermediary Inter-Organizational Perspective*, Health Pol. (2017).

25 D. Valeeva, E.M. Heemskerk & F.W. Takes, *The Duality of Firms and Directors in Board Interlock Networks: A Relational Event Modeling Approach*, Soc. Netw. 2 62 (2020).

26 J.S.G. Chu & G.F. Davis, *Who Killed the Inner Circle? The Decline of the American Corporate Interlock Network*, Am. J. Soc. (2016).

27 Val Burris, *Interlocking Directorates and Political Cohesion among Corporate Elites*, Am. J. Soc. (2005).

28 L. Qu, Y. Xu & Y. Guo, *Research on the Influence of Network Position on Corporate Social Responsibility: Moderating Effect Based on Ownership Concentration*, Front psychol. (2022).

29
P. E. Areeda, H. Hovenkamp, Antitrust Law: An Analysis of Antitrust Principles and Their Application¶1300 (4th ed. 2016).

30
https://www.justice.gov/opa/speech/assistant-attorney-general-jonathan-kanter-delivers-opening-remarks-2022-spring-enforcers (accessed Mar. 24, 2024).

31
https://www.ftc.gov/enforcement/competition-matters/2019/06/interlocking-mindfulness (accessed Mar. 24, 2024).

32
https://www.justice.gov/opa/pr/directors-resign-boards-five-companies-response-justice-department-concerns-about-potentially (accessed Mar. 24, 2024).

33 L. Kaplow, *Why (Ever) Define Markets?*, Harv. Law Rev. 124, 438 (2010).

34 Clayton Antitrust Act §8, 15 U.S.C. §19.

35 FTC Announces Annual Update of Size of Transaction Thresholds for Premerger Notification Filings and Interlocking Directorates, Federal Trade Commission, (2022). https://www.ftc.gov/news-events/news/press-releases/2022/01/ftc-announces-annual-update-size-transaction-thresholds-premerger-notification-filings-interlocking (accessed Mar. 24, 2024).

36 P. Lehoux, F.A. Miller & G. Daudelin, *How Does Venture Capital Operate in Medical Innovation?,*  Bmj Innov. (2016).

37 M.A. Carrier, M.A. Lemley & S. Miller, *Playing Both Sides: Branded Sales, Generic Drugs, and Antitrust Policy*, Hast. Law J. 71, 307 (2020).

