# Supplementary material for: Illegal interlocks among life science company boards of directors
Source: J Law Biosci. 2024 Apr 13;11(1):lsae005. doi: 10.1093/jlb/lsae005 (PMC11017978; doi:10.1093/jlb/lsae005)
Supplement: Supplementary_data_lsae005 [file supplementary_data_lsae005.docx]

**Supplementary Figures**


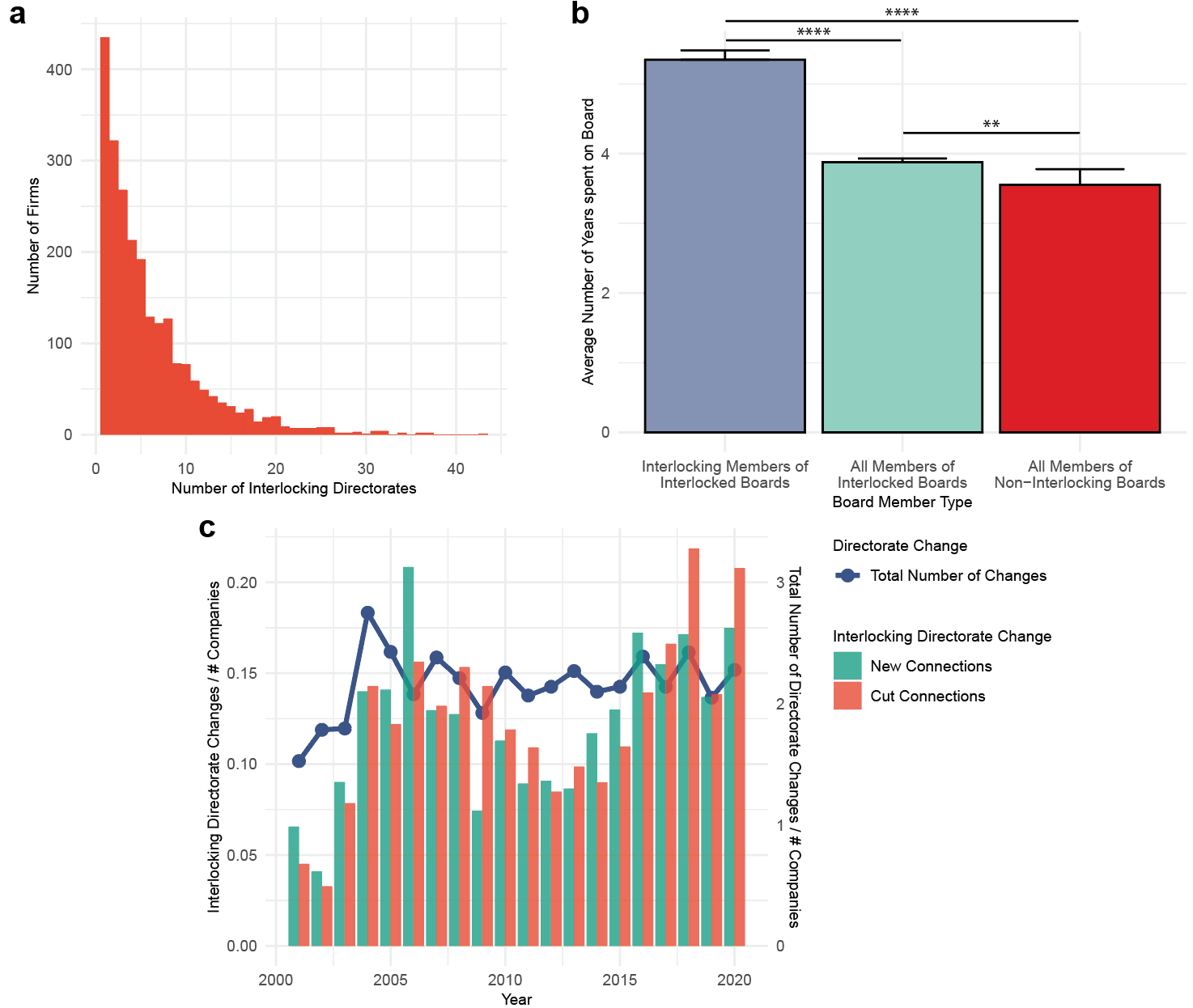


**Supplemental Figure 1:** (A) Histogram of network shown in Figure 1B illustrating the number of firms that have had the corresponding number of interlocking directorate events since 2001. (B) Comparison of the average tenure of board members depending on whether they were an interlocking member of a board, were a member of a board of a firm that had an interlocking board, or were a member of a board that did not interlock (** indicates p < 0.01, **** indicates p < 2.2e-16) (C) Number of interlocking directorates formed and dissolved between companies (left y-axis) and total number of directorship changes (right y-axis) annually from 2001-2020 normalized by the number of companies in each year.


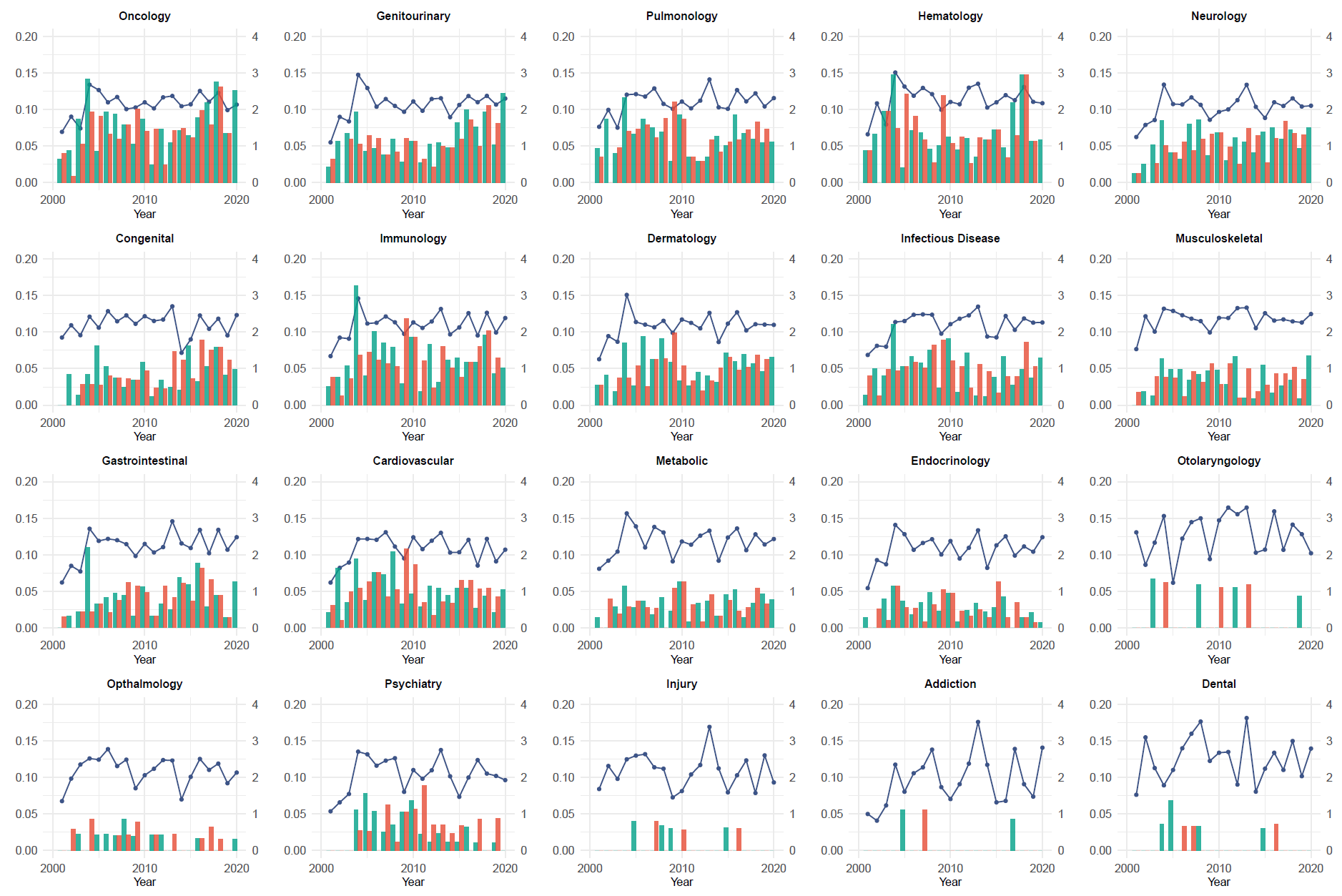


**Supplemental Figure 2:** Number of interlocking directorates formed and dissolved between companies (left y-axis) and total number of directorship changes (right y-axis) annually from 2001-2020, by the disease categories of clinical trial indications they have sponsored normalized by number of companies in the field and year.


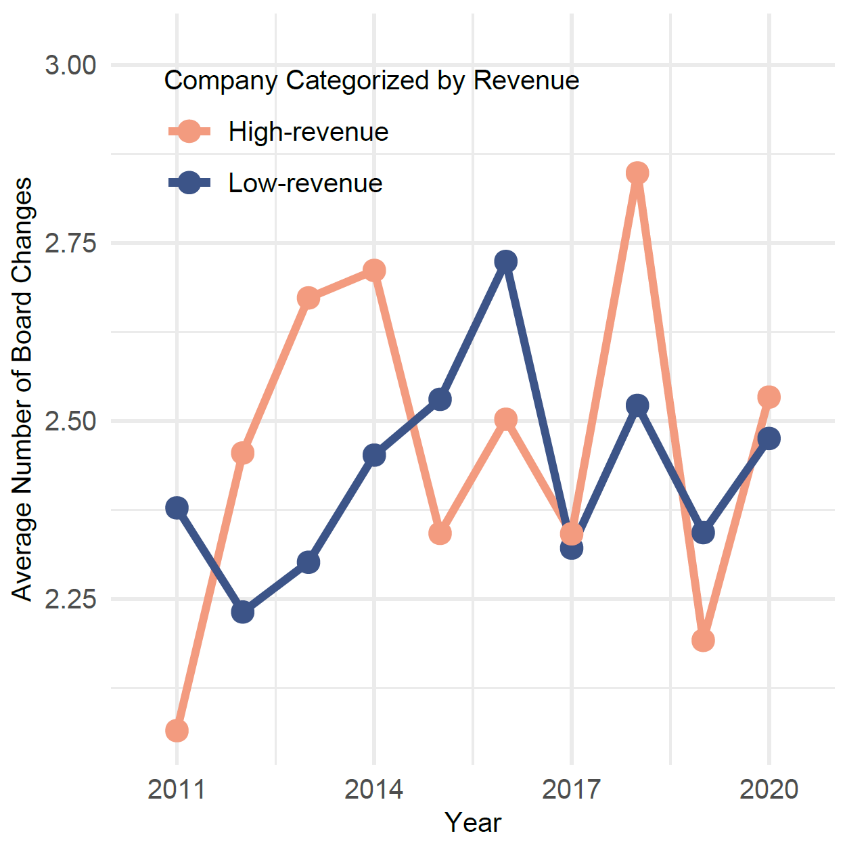


**Supplemental Figure 3:** Average number of board changes for companies with greater than or less than $5 million in historical revenue from 2011-2020.

**Methods**

In this work we explore the dynamics of the board of directors of firms working in the biological sciences. Considering all industry sponsors of clinical trials from ClinicalTrials.gov, we isolated those with public filings with the Security and Exchange Commission (SEC). We then extracted board membership information from these filings for each year.

**ClinicalTrials.gov Data**: In order to focus our research on firms engaged in biological and clinical work we identified all industry sponsors of clinical trials from ClinicalTrials.gov. Public data on all clinical trials in ClinicalTrials.gov, which includes industry sponsor information, is available free to all researchers at <https://clinicaltrials.gov/AllPublicXML.zip>

**SEC Public Filing Data:** Data on firm directorates as well as revenue data were isolated from firm 10-Ks, which are mandatory annual disclosures of certain financial data and board membership. A database of all public SEC filings is available free to all researchers at <https://www.sec.gov/Archives/edgar/daily-index/bulkdata/submissions.zip>.

**Firm Filing Data:** We first downloaded all publicly filed 10-K forms by searching through the index of all public SEC filings at <https://www.sec.gov/Archives/edgar/daily-index/bulkdata/submissions.zip> for all entries corresponding to a 10-K form. The CIK code, firm name, and 10-K link were recorded, and the 10-K html files downloaded from the link. Implementation details for extracting this information is provided at *10-K.py*

**Revenue Data:** Revenue data was determined for all companies using the Intrinio equities API, specifically the fundamentals dataset. The CIK of each company in the database was used as an identifier to retrieve the “totalrevenue” datapoint, representing the total revenue reported, for each year between 2000 and 2020. Further implementation details are included in *revenue.py*

**Identifying firms of interest:** We isolated all sponsors of clinical trials listed as industry from the ClinicalTrials.gov public database. In order to perform directorate analysis, the name of each sponsor needed to be matched to a corresponding entry in the SEC database. To this end we first attempted direct matching between sponsor names and those in the 10-K database created in the last step.

Next, elements of the string were replaced or removed to improve matching. For instance, “corporation” and “corp” were removed, “pharmaceuticals” was shortened to “pharma”. A dictionary of elements of the string that were matched, and their replacement is provided in *clin.py*. Name strings were also normalized by setting all characters to lower case, removing punctuation as well as excess white space. Sponsor matching to SEC names was attempted once more with this approach.

For sponsors that remained unmatched, the first word of each firm was extracted. For firms with generic first words (e.g. “The”, “American”, “Cardiac”), the first two words were extracted. These keywords were then used to search for firms on the SEC Edgar full text search ([SEC.gov | EDGAR Full Text Search](https://www.sec.gov/edgar/search/)). The CIK codes of the resulting firms were then aggregated for each keyword. A potential concern with this approach is the chance for spurious matches. Each company is associated with a SIC code that indicates its rough industry of operation. In order to reduce spurious matches, only CIK codes corresponding to SIC codes managed by the SEC’s Office of Life Science were considered. These SIC codes include the following:

["100", "200", "700", "800", "900","2800", "2810","2820", "2821", "2833", "2834", "2835", "2836", "2840", "2842", "2844", "2851", "2860", "2870", "2890", "2891", "3821", "3822", "3823", "3824", "3825", "3826", "3827", "3829" ,"3841", "3842", "3843", "3844", "3845", "3851", "3861", "3873", "8000" ,"8011", "8050", "8051", "8060", "8062", "8071", "8082", "8090", "8093"]

Once the list of CIK codes corresponding to each keyword was enriched using this method, the CIK code with the most number of entries from the search was considered to correspond to the sponsors associated with the keyword.

Further implementation details are provided in *clin.py*

**Field Matching:** In order to analyze directorate changes across different fields of medicine each firm had to be assigned a field of medicine. This was done using clinical trial indication. All the clinical trials indications for each unique sponsor were aggregated. ClinicalTrials.gov has a mapping of indication to category available [here](https://clinicaltrials.gov/ct2/results/browse?brwse=cond_cat). Firms were assigned a field if they had at least one clinical trial with at least one indication associated with the field. Mapping of ClinicalTrials.gov category to field is provided below:

Behaviors and Mental Disorders: Psychiatry

Blood and Lymph Disorders: Hematology

Digestive System Diseases: GI

Diseases or Abnormalities at or Before Birth: Congenital

Ear, Nose, and Throat Disease: ENT

Eye Diseases: Ophthalmology

Gland and Hormone Related Diseases: Endocrinology

Heart and Blood Diseases: Cardiovascular

Immune System Diseases: Immunology

Infections: Infectious Disease

Mouth and Tooth Disease: Dental

Musculoskeletal Diseases: MSK

Neoplasms: Oncology

Nervous System Diseases: Neurology

Nutritional and Metabolic Diseases: Metabolic

Respiratory Tract (Lung and Bronchial) Diseases: Pulmonology

Skin and Connective Tissue Diseases: Dermatology

Substance Related Disorders: Addiction

Urinary Tract, Sexual Organs, and Pregnancy Conditions: GU

Wounds and Injuries: Injury

Implementation details are provided in *clin.py*

**Board Extraction**: The names and roles of board members were extracted from the signature page of 10-K forms. The signature page includes the symbol “/s/” representing each member’s signature, followed closely by the board member’s name and position. The text between each “/s/” was extracted and a regex expression was used to isolate the board member’s name and position (officer or director). Implementation details are provided in *extraction.py*

**Name Cleanup and Disambiguation:** Board members occasionally use slightly different variations of their name between years and companies. In order to track board membership across time and track individuals between companies it is necessary to disambiguate names.

First all names were cleaned by removing punctuation, accents, and extraneous white space. Each unique combination of first name, middle initial, and last name were found. Redundancies were determined for middle names (people with unique combinations of first and last name, meaning that the middle name did not provide distinguishing information), and the middle names for these individuals were removed. If the first name past the first initial failed to provide additional distinguishing information, only the first initial was included.

Implementation details are provided in *name_disambig.py*

**Revenue Categorization:**

Firms were categorized as either having or not having “large” revenue, which was defined as $5 million over a year. Company revenue status was determined on a yearly basis. Once a company was considered to have “large” revenue it was considered to have a “large” revenue for the duration of the dataset

**Determination of Interlocking Directorates:** Two firms were considered to have interlocking directorates should an individual be listed as a board member of both firms during the same fiscal year.

For the purpose of calculating average number of interlocking directorates, each individual that was interlocking was considered to be interlocking on each firm (e.g. a director interlocking firm A and B was counted once for firm A and once for form B). The ribbon in figure 1d represents the 95% confidence interval of the mean number of interlocking directors.

**Visualization of Whole Biology Network:** The visualization in 1b represents the shared board members of all firms in biology between 2000 and 2020. Each node represents a company and each edge indicates that at least one board member was shared between the companies. The nodes are colored from blue to red with blue representing fewer connecting nodes and red representing more connecting nodes. The graph was produced in Gephi version 0.9.2 using a Fruchterman Reingold layout with default parameters.

**Visualization of network by time/field:** The visualizations in figures 1c and 2b illustrate interlocking directorates for each year and field respectively.

In 1c, each node represents a company for a given year and an edge represents a shared board of director for that year. Nodes are again colored from blue to red with blue representing fewer interlocked directors for the year and red representing more interlocked directors for the year.

2b was produced similar to 1c with the nodes representing companies that have at least one clinical trial with an indication in the given field and edges between companies that shared at least one director between 2000 and 2020. Nodes are again colored from blue to red with blue representing fewer interlocked directors for the year and red representing more interlocked directors over the time period.

Both figures were produced with Python Plotly version 4.2.1 with the graphviz layout function from the network package version 2.8.4

**Interlocked Directorates by Indication:** Figure 2a illustrates the number of interlocked companies within a clinical trial indication between 2000 and 2020. A company was considered to be part of an indication should it be listed as a sponsor of a clinical trial with the given indication. The number of companies in each indication that shared a board member was summed over the years 2000-2020 to generate the total number of interlocking boards for the indication.

**Interlocked boards between Competitors:** In order to provide a gold standard for competitors, we considered a company’s competitors that it directly listed on their financial disclosures to the SEC. Docoh (<https://docoh.com/>), a filings aggregator was utilized to identify each company’s explicitly listed competitors. Each company’s profile page on Docoh was scraped for the list of its competitors. Each pair of competitors had their boards analyzed for interlocking, with the number of interlocking boards between explicitly listed competitors provided for each year between 2000 and 2020.

**Determination of Directorate Change:** The rate of change in board membership was determined both in terms of raw number of changes as well as changes to interlocking directorates. The overall change in board membership was calculated by finding all companies for a given year (and field) were present in the dataset during the previous year. The number of change (both new additions as well as departures) was summed for all companies to produce the overall number.

To track changes in interlocking directorates, companies that were present in the data in both the year under consideration as well as the previous year were isolated. A new connection was considered to be made if a director on a firm in a previous firm became a director on a new firm. A connection was considered to be cut should a director on two or more firms was no longer a director on a firm.

Implementation details are included in *network_vis.py*

**Change in Directorate Between Revenue Groups:** We hypothesized that the velocity of directorate changes differed between companies with more than $5 million in annual revenue and companies that did not. To this end, we categorized companies by their revenue and found the average change in board seats over time. The number of change (both new additions as well as departures) was summed for all companies to produce the overall number.

We also investigated the change in the network of interlocking directors. Companies that were present in the data for the year under consideration as well as the previous year were categorized by revenue status. Interlocking directors were determined to be either between high revenue firms, low revenue firms or between high and low revenue firms. The number of changes to these interlocking directors was calculated for those interlocking high revenue, low revenue, and a mix of high and low revenue firms. We then presented the average change in the interlocking directorates by type of interlocking director in figure 3e. We also aggregated the number of changes from 2001-2020 and present this in figure 3f. Error bars represent the 95% confidence interval.
